# Local Sociocultural Practice Specific Financial Toxicity Assessment Tool for Cancer: An Emerging Need

**DOI:** 10.1200/GO.24.00039

**Published:** 2024-03-28

**Authors:** Parth Sharma, Muttacaud Ramakrishnan Rajagopal, Enrique Soto-Perez-de-Celis, Charmaine Blanchard, Deepak Sudhakaran, Aju Mathew

**Affiliations:** Parth Sharma, MBBS, Association for Socially Applicable Research (ASAR), Pune, India, Department of Community Medicine, Maulana Azad Medical College, New Delhi, India; Muttacaud Ramakrishnan Rajagopal, MD, MBBS, Trivandrum Institute of Palliative Sciences, Trivandrum, India; Enrique Soto-Perez-de-Celis, MD, PhD, FASCO, Departamento de Geriatría, Instituto Nacional de Ciencias Médicas y Nutrición Salvador Zubirán, Mexico City, Mexico; Charmaine Blanchard, MBBCh, MPhil, PD Dip Pall Med, Centre for Palliative Care, Faculty of Health Sciences, University of the Witwatersrand, Johannesburg, South Africa, Strengthening Oncology Services Research Unit, Faculty of Health Sciences, University of the Witwatersrand, Johannesburg, South Africa; Deepak Sudhakaran, MD, MBBS, Trivandrum Institute of Palliative Sciences, Trivandrum, India; and Aju Mathew, MD, DM, MBBS, MPhil, MOSC Medical College, Kolenchery, India

## Abstract

It is relevant to study the financial toxicity of cancer to address it. However, the existing tools fail to capture the financial destruction of cancer on patients and their families in resource-limited countries. The authors discuss the need for a new tool in this article.

## To the Editor:

The burden of cancer has been rising globally with lower-middle and lower sociodemographic index countries being disproportionately affected.^1^ It is well known that cancer treatment is not only long in duration but also expensive. It therefore can push people into poverty, especially in low- and middle-income countries (LMICs) where out-of-pocket health care expenditure (OOPE) is high. ^2^ Realizing this, researchers have started focusing on the financial aspect of cancer care. Over recent years, researchers from across the world have studied the financial toxicity (FT) of cancer either by quantifying the OOPE or by doing a subjective assessment using questionnaires.^3^

Assessment of catastrophic health expenditure and impoverishment is the ideal way to assess the FT of cancer treatment. However, because of a lack of expertise in health economics, researchers often rely on subjective questionnaires to understand the financial destruction caused by the disease. The commonly used tools specifically for cancer include the following:

1. The Comprehensive Score for Financial Toxicity (COST),^4^
2. Socioeconomic Wellbeing Scale (SWBS),^5^ and
3. Breast Cancer Finances Survey Inventory (BCFS).^6^

However, these tools were developed and validated by researchers in the United States, a high-income developed nation. A systematic review by Witte et al^3^ highlighted that even self-designed questionnaires have mostly been used only in high-income countries to study the FT of cancer.

The COST, a self-administered questionnaire, consists of 12 questions of which 11 are used to measure FT. The questions focus on financial spending (one question), financial resources (two questions), and the psychosocial response to the financial hardship of cancer patients (eight questions). SWBS assesses FT using 17 questions divided into two subscales: material (nine items) and social capital (eight items). The material subscale assesses the financial expenditure and resources, and the social capital subscale assesses the psychosocial resources regarding family support or the person's health literacy. Finally, the BCFS contains 42 questions, with 19 focusing on changes in work and financial hardship. Questions on work assess motivation, productivity, quality of work, and absenteeism from work, and those on financial hardships assess change in income, lifestyle, and insurance coverage.

However, these questions fail to address the sociocultural factors prevalent in LMICs such as India, Mexico, and South Africa among others. As collective autonomy is practiced in India, it becomes difficult for the patient to answer questions on FT alone.^7^ The COST questionnaire therefore might give falsely low FT if the family members do not discuss the financial situation with the patient and falsely high if the patient perceives themselves to be a burden on the family. The COST tool becomes further ineffective in assessing FT if the patient has never been employed and never made any financial decisions in the family. Patriarchal culture could also result in a falsely low assessment of FT if the tool does not contain the right questions to assess the same, specifically for women.^8^ Similarly, SWBS and BCFS too fail to capture the depth of the FT of cancer, especially in the context of LMICs.

Cancer affects the entire family and not the person alone. Therefore, a tool for assessing FT needs to look beyond debt and loss of assets because of and needs to assess common region-specific experiences.^9^ An ideal tool should be easy to interpret and understand by the patient and should be able to correctly identify the people affected most by the financial destruction due to the disease.

Loss of employment or wages of caregiver, decrease in food consumption due to unaffordability, discontinuation of education of any family member, negative impact on the treatment of any other family member's illness, and history of discontinuation of treatment or history of seeking alternate forms of care because of lack of financial resources could be included as a part of the tool as these are the common difficulties that cancer affected patients and families face. To add a more humane touch to it, the tool could also assess if the families had stopped celebrating their key cultural events because of financial restrictions after the patient's cancer diagnosis. The psychosocial aspect of financial destruction could also assess the loss of family or social support after the cancer diagnosis. ^10^ The status of women in society is variable in different countries, and FT may hurt them more than men. When one member of the family falls ill, in a patriarchal society it is often the women who are expected to leave their jobs or to discontinue education to care for the diseased. A tool to assess FT would need to explore its gender-based impact too.

Ultimately, the entire family unit needs to be assessed to understand the FT of the disease including its impact on subsequent generations and not the patient alone. This will help in modifying social security schemes and help in making cancer treatment less toxic.

## References

[1] Global Burden of Disease 2019 Cancer Collaboration, Kocarnik JM, Compton K, et al: Cancer incidence, mortality, years of life lost, years lived with disability, and disability-adjusted life years for 29 cancer groups from 2010 to 2019: A systematic analysis for the global burden of disease study 2019. JAMA Oncol 8:420-444, 2022 34967848 10.1001/jamaoncol.2021.6987 PMC8719276

[2] Donkor A, Atuwo-Ampoh VD, Yakanu F, et al: Financial toxicity of cancer care in low- and middle-income countries: A systematic review and meta-analysis. Support Care Cancer 30:7159-7190, 2022 35467118 10.1007/s00520-022-07044-z PMC9385791

[3] Witte J, Mehlis K, Surmann B, et al: Methods for measuring financial toxicity after cancer diagnosis and treatment: A systematic review and its implications. Ann Oncol 30:1061-1070, 2019 31046080 10.1093/annonc/mdz140 PMC6637374

[4] de Souza JA, Yap BJ, Hlubocky FJ, et al: The development of a financial toxicity patient-reported outcome in cancer: The COST measure. Cancer 120:3245-3253, 2014 24954526 10.1002/cncr.28814

[5] Head BA, Faul AC: Development and validation of a scale to measure socioeconomic well-being in persons with cancer. J Support Oncol 6:183-192, 2008 18491687

[6] Given BA, Given CW, Stommel M: Family and out-of-pocket costs for women with breast cancer. Cancer Pract 2:187-193, 1994 8055022

[7] Sutar R, Chandra PS, Seshachar P, et al: A qualitative study to assess collusion and psychological distress in cancer patients. Indian J Palliat Care 25:242-249, 2019 31114111 10.4103/IJPC.IJPC_146_18 PMC6504745

[8] Alexander A, Kaluve R, Prabhu JS, et al: The impact of breast cancer on the patient and the family in Indian perspective. Indian J Palliat Care 25:66-72, 2019 30820105 10.4103/IJPC.IJPC_158_18 PMC6388591

[9] Sánchez-Román S, Chavarri-Guerra Y, Vargas-Huicochea I, et al.: Financial toxicity among older Mexican adults with cancer and their families: A mixed-methods study. JCO Glob Oncol 10.1200/GO.21.00324 10.1200/GO.21.00324 PMC8932483 35286137

[10] Mlaba PC, Ginindza TG, Hlongwana KW: The social burden experienced by families caring for members living with cancer in KwaZulu-Natal, South Africa. Afr J Prim Health Care Fam Med 13:e1-e10, 2021 10.4102/phcfm.v13i1.2955 PMC8603144 34797113

